# Influence of Freeze Concentration Technique on Aromatic and Phenolic Compounds, Color Attributes, and Sensory Properties of Cabernet Sauvignon Wine

**DOI:** 10.3390/molecules22060899

**Published:** 2017-06-02

**Authors:** Yan-Yan Wu, Kai Xing, Xiao-Xu Zhang, Hui Wang, Yong Wang, Fang Wang, Jing-Ming Li

**Affiliations:** 1 Center for Viticulture and Enology, College of Food Science and Nutritional Engineering, China Agricultural University, P.O. Box 301, Beijing 100083, China; wyyfendou@163.com (Y.-Y.W.); zxxjoypeace@foxmail.com (X.-X.Z.); 2 Sino-French Joint Venture Dynasty Winery Ltd., Tianjin 300402, China; kyle.xk@139.com (K.X.); d-dahui@163.com (H.W.); wywy163@126.com (Y.W.); angelawangfang@yeah.net (F.W.)

**Keywords:** freeze concentration, HS-SPME-GC-MS, HPLC-MS/MS, aromatic volatiles, phenolic compounds, wine

## Abstract

Red wines produced in the Xinjiang region of China possess poor color density, and lack fruity notes and elegance. The freeze concentration technique, as a well-established concentration method for liquid food systems, was applied to the Cabernet Sauvignon (*Vitis vinifera* L.) wine-making process, aiming to investigate its effect on wine quality improvement. Results showed that the freeze concentration treatment did not significantly alter the physicochemical properties of the wine, except for an increase of glycerol and alcoholic content. This technique increased ester contents, as well as decreasing the amount of volatile acids. Higher alcohol contents were also increased, but within an acceptable content range. All taken into consideration, the freeze concentration treated wine showed better fragrance characters according to sensory evaluation. The non-anthocyanin composition was altered by this application, however, the difference disappeared after the aging process. Fortunately, sensory evaluation showed that the treated wine possessed better mouthfeel properties. Anthocyanin contents were enhanced, and effectively stabilized the fresh wine color attributes, resulting in an improvement in appearance of the treated wine. All results considered, it can be concluded that freeze concentration treatment could be a good choice to improve wine quality.

## 1. Introduction

Aromatic and phenolic compounds are the crucial compounds that determine the quality of wine, and their evolution during the wine-making process affects both wine feature and maturation [1,2]. Wine aroma is extremely complex. Some of the aromatic compounds are derived from grape berries, and they exhibit fruity notes [3]. The fermentation process results in the formation of fermented aromatic compounds in wine [4]. Moreover, the aromatic compounds with aging aroma notes are normally synthesized in wine during the aging process, which further helps wine maturation [5]. Aroma is usually the first choice for consumers [6]. It is reported that the consumer acceptability of a wine is generally determined by whether or not it contains a complex but well-balanced aroma profile [7]. Phenolic compounds are mainly extracted from grape berries into wine during the fermentation process, and their evolution alters the color, mouthfeel, and palatability of the wine [8]. Anthocyanins play an important role in the wine color attributes, whereas non-anthocyanin phenolic compounds determine the astringency and bitterness of wine [9]. It is of great importance to enhance the aromatic and phenolic contents in wine to improve wine quality.

The freeze concentration technique is a well-established concentration method for liquid food systems. It basically includes three steps: ice crystal formation, growth, and removal. The aim of the technique is to reduce the water content by removing the ice crystals, and then to increase the target components in the liquid products [10]. Considering that this concentration process takes place in a low temperature environment, the products still preserve their initial organoleptic properties [11]. Freeze concentration is widely utilized in the food industry, such as for fruit juice and dairy production to improve the nutrient content [10,11]. However, its application in the wine-making process has been limited. In the existing study, the application of freeze concentration in vinification was mainly to increase the insufficient sugar content of the grape, from 14°Brix, the initial sugar content, to 23°Brix [12].

The Xinjiang region in China belongs to an arid and semi-arid climate with a pretty low annual rainfall amount, which makes it ideal for viticulture [13,14]. Although the high day temperature and intense sun exposure provide grapes with sufficient sugar content, some secondary metabolites in red grapes can be insufficient, causing red wine with a poor color density and stability and a lack of fruity aroma [14,15]. In this study, we selected *Vitis vinifera* L. Cabernet Sauvignon grapes grown in the Xinjiang region from a 2014 vintage, and applied the freeze concentration technique during the wine-making process. The freeze concentration treated wines were compared with the traditionally fermented wines (the control) in terms of their aromatic and phenolic compositions, color attributes, and sensory features after fermentation and aging periods. The findings from this study provide useful information on the application of freeze concentration technique on wine production development.

## 2. Results and Discussion

### 2.1. Physicochemical Index

In order to monitor the effect of the freeze concentration process on the juice composition, the physicochemical indices of the fresh juice from the grape berries, the concentrated juice after freezing, and the mixed juice to be fermented, as well as the removed portion were determined (Table 1). The sugar contents were significantly increased in the concentrated juice and the mixture. The mixed juice reached a final sugar content of 255.71 g/L, possessing a potential alcohol content of 14% approximately. In the removed portion, a low content of sugar was detected, because the practical operation could not obtain absolute pure ice [10,16]. Total acid and pH were not significantly changed after the treatment, which was consistent with Belén [17]. Volatile acid was detected in a very low level in these samples, especially in the removed portion.

After fermentation, the wine with the freeze concentration treatment basically had similar physicochemical properties to the control within an acceptable range [18]. It demonstrated that the freeze concentration technique did not change the basic properties of the Cabernet Sauvignon wine-making process significantly (Table 2).

The alcohol content was increased by approximately 1 vol % (*p* ≤ 0.05) in treated wine because of the concentration technology. The content of glycerol in the treated wine was also significantly higher than control; this may be related to the yeast metabolism. Under hyperosmotic conditions, yeasts have to produce osmoprotectant to protect themselves; while glycerol is a good osmoprotectant synthesized as a by-product of alcoholic fermentation [19]. Freeze concentration causes a hyperosmotic stress for yeasts because of the higher sugar content, so yeasts generate more glycerol during fermentation, and thus a higher content of glycerol was produced in the treated wine.

### 2.2. Volatile Compounds

A total of 81 aromatic compounds (Table 3) were detected in these wines, among them 16 volatile compounds (highlighted in bold font) which exhibited an important contribution to the overall aroma of the wine due to their concentration being higher than the threshold (Odor activity values (OAV) above 1.0).

Esters are secondary metabolites from yeast, synthesized via higher alcohols and acetyl-CoA metabolism [20,21]. They constituted the largest group of volatiles, and contribute the fruity and flowery characters of wine aroma [20]. In this study, in all 33 esters were detected. After the fermentation, most of the esters showed higher concentrations in the treated wine compared with the control (*p ≤* 0.05). Only a few of them were higher in the control than in the treated wine, but their contents were all below the threshold. Ethyl acetate, ethyl butanoate, isoamyl acetate, ethyl hexanoate, ethyl octanoate, isoamyl lactate, ethyl decanoate, and ethyl dihydrocinnamate exhibited a content above the threshold (OAV > 1.0) and showed a higher content in the treated wine, which implied an improved fruity aroma could be obtained after freeze concentration.

Higher alcohols, also called fusel alcohols, are produced by yeast during alcoholic fermentation [22], and usually have a strong and pungent smell, as well as taste. Total higher alcohols in the samples were all in the acceptable range 400–1400 mg/L for red wines [23]. However, the content was significantly higher in the treated wine than the control (*p* ≤ 0.05) as a consequence of the high ethanol content and high level of pomace to juice ratio, in agreement with Fleet and Heard. Fleet and Heard found ethanol concentration and solids content could affect the higher alcohols concentration in the final product [24]. 1-Propanol, isobutanol, 1-butanol, isopentanol, and 1-hexanol were the dominant higher alcohols in these wines, and also showed higher content in the treated wine. However, only isopentanol and 1-hexanol showed higher concentrations than the threshold (OAV > 1.0); these two higher alcohols contribute solvent and herbaceous notes for wine, respectively. The OAV of 2-phenylethanol was also above 1.0. Unlike other aliphatic alcohols, 2-phenylethanol is an aromatic alcohol and contributes to rose or honey characters [25]. However, in our study, freeze concentration technology showed a negative influence on this compound. The reason why the treated wine showed a lower amount of 2-phenylethanol is not clear, and further studies are needed.

Volatile acids belong to a type of odorant compound with unpleasant smells. These compounds generally possess pungent, rancid or fatty characters. Seven volatile acids were detected in the treated and control wines, including acetic acid and six fatty acids. The content of acetic acid in treated wine was significant lower than the control, and also the total fatty acid content. Among them, hexanoic acid and octanoic acid had a content above the threshold. Most of the volatile acids showed lower content in the treated wine than the control. The results implied that the aroma character of the treated wine could be more acceptable than the control.

Terpenes are another series of volatiles contributing pleasant aromatic notes to wine. They are important floral-related compounds and usually illustrate the variety feature of a wine. Normally, terpenes exist in grape berries as their glycosidic forms and these glycosidic conjugated terpenes are flavorless [38]. These glycosidic conjugated terpenes are able to be hydrolyzed by enzymes during the wine-making process to release aglycones, which enhance the varietal characters [38]. In this study, among the eight detected terpenens, geraniol, nerol, and β-damascenone had a concentration above the threshold. However, all the terpenes showed similar contents in the treated and control wines, indicating that the freeze concentration treatment had little influence on terpenes.

After aging for one year, the ester contents changed differently, and their total amount was significantly higher in the treated wine than the control (*p* > 0.05). It is reported that both esterification and hydrolysis reactions could occur during aging [39], therefore the increase or decrease of esters could simultaneously appear in wine. The evolution of esters during aging is different, depending on their structures. Generally, the ethyl branched acid esters increase, while ethyl fatty acids esters and acetate of higher alcohols decrease [40,41]. In this study, ethyl branched acid esters, such as ethyl benzoate and ethyl phenylacetate increased, while, acetates of higher alcohols, such as isoamyl acetate, hexyl acetate and phenethyl acetate decreased. It is worth mentioning that the total amount of esters remained higher in the treated wine, indicating that the freeze concentration treatment could benefit the enhancement of esters. Terpenes and C_13_-norisoprenoid also changed in different ways during aging. Linalool showed an increase, while β-citronellol, geraniol, β-damascenone, and geranylacetone decreased significantly (*p* ≤ 0.05). These compounds showed a similar content in the treated and control wines after aging. Combining the evolution of esters and terpenes, it is speculated that freeze concentration treatment could enhance the fruity notes of a young wine, however the enhancement could be diminished by the aging process.

Most of the higher alcohols remained stable during aging, and their total amount was significantly higher in the treated wine than the control (*p* ≤ 0.05). This could be unfavorable for the aroma elegance or harmony [15]. On the other hand, the volatile acids showed significantly lower content in the treated wine (*p* ≤ 0.05), indicating freeze concentration would reduce the rancid or fatty character notes of the wine [4]. This could be favorable for the aroma quality. Based on the change of higher alcohols and volatile acids, overall evaluation by sensory panels is needed to determine how freeze concentration can influence the wine aroma quality.

### 2.3. Phenolic Compounds

#### 2.3.1. Non-Anthocyanin Phenolic Compounds

A total of 32 non-anthocyanin phenolic compounds were detected in the wines, including seven flavan-3-ols, 12 flavonols, eight phenolic acids, and five stilbenes (Table 4). Both of the wines showed the highest content for the total flavan-3-ols, followed by flavonols and then phenolic acids. Stilbenes showed the lowest content in these wines. These results were consistent with a previous report [42]. The freeze concentration treatment did not significantly alter the total content of the non-anthocyanin phenolic compounds compared with the control (Table 4). These non-anthocyanin phenolic compounds play different roles in affecting the sensory attributes and quality of wine. Therefore, it is quite critical to investigate the effect of the freeze concentration treatment on individual non-anthocyanin phenolic compounds in the wine.

After the fermentation process, the significantly higher contents of catechin and epicatechin (Table 4) resulted in a 20% increase on the total flavan-3-ols content for the treated wine compared with the control. The phenomena were possibly related to the higher alcoholic strength in freeze concentration treated wine than the control (Table 2), since it has been reported that an increase in ethanol percentage in wine increased the extraction of flavan-3-ols into the wine [43]. Flavan-3-ols mainly contribute to wine with astringency and bitterness effects, and thus the treated wine after the fermentation might exhibit better astringency and bitterness properties [42]. Therefore, it was expected that the freeze concentration application could benefit the mouthfeel of wine due to its higher content of flavan-3-ols. After one-year aging, the content of flavan-3-ols changed to a similar level in the treated and control wines (Table 4). This might be because t oxidation might occur with flavan-3-ols during the aging period [9]. Besides, their copigmentation with anthocyanins could also result in the decrease of flavan-3-ols [44].

A total of 12 flavonols were detected after the wine fermentation. Quercetin and its glycosides appeared to be the predominant flavonols (Table 4). The result was consistent with a previous report [45]. The freeze concentration treatment did not significantly affect the total content of flavonols in the wines after fermentation (Table 4). However, some individual flavonols were observed to be different between the treated and the control wines. For example, quercetin-3-*O*-rutinoside and quercetin-3-*O*-rhamnetin were only detected in the treated wine. The treated wine contained higher content of kaempferol-3-*O*-glucoside, but lower content of quercetin, taxifolin, and isorhamnetin. After one-year aging, the content of flavonols in the treated wine was similar to that in the control except that higher contents of kaempferol and isorhamnetin were observed (Table 4). Glycosidic conjugated flavonols showed a content decrease after one-year aging, whereas an increase in their aglycones was observed. The decrease of flavonol glycosides was mainly due to condensation, oxidation, and copigmentation reactions [46], as well as hydrolysis of the glycosides to produce corresponding aglycones [47]. Particularly, the copigmentation of flavonols with anthocyanins has been reported to result in color enhancement of wine after aging, since the copigmented anthocyanins have a more stable structure [44,45]. Flavonols also provide bitterness and astringency to wine [48]. However, the freeze concentration treatment did not favor the flavonol contribution to wine quality due to its negligible influence on flavonol contents.

These wines after the fermentation process contained eight phenolic acids, including three hydroxycinnamic acids and five hydroxybenzoic acids (Table 4). These phenolic acids existed in the wines at a low content. Hydroxycinnamic acids can serve as strong antioxidants and can be oxidized to yield brown pigments [48]. Besides, they also play important roles in stabilizing anthocyanins and improving the color of wine [48]. After the fermentation, gallic acid appeared to be the predominant hydroxybenzoic acid in these wines, and its content was higher in the control (Table 4). Similarly, caffeic acid was also observed in a higher content in the control. After one-year aging, hydroxycinnamic acids showed a content increase, whereas a decrease in the content of hydroxybenzoic acids was observed (Table 4). As a result, caffeic acid appeared to be the predominant phenolic acid in these aged wines. The increase of hydroxycinnamic acids could be caused by the hydrolysis of esters during aging [46]. Besides, these molecules can also participate in polymerization and/or oxidation [9,49]. Hydroxybenzoic acids, like gallic acid, also experience oxidation, esterification, and/or acetylation during aging, resulting in content decrease [46,50].

Stilbenes have been confirmed to show health-promoting benefits in wine [42]. In the present study, a total of five stilbenes was detected in the treated and control wines (Table 4). There was no consensus on stilbene evolution during aging, since both increases and decreases of them have been reported in different studies [42,51]. The stilbenes in our study showed significant decrease during aging, and no significant content difference was found between the treated and control wines. Therefore, freeze concentration treatment did not affect the content of stilbenes in wine.

#### 2.3.2. Anthocyanins and Color Attributes

The freeze concentration treatment showed a similar anthocyanin profile as the control (Table 5). Regarding their structures, these anthocyanins can be grouped into four categories: non-acetylated anthocyanins, acetylated anthocyanins, pyranoanthocyanins, and polymeric anthocyanins. Both of the wines had non-acetylated anthocyanins as their predominant anthocyanins, followed by the acetylated anthocyanins, pyranoanthocyanins, and then polymeric anthocyanins.

After fermentation, the treated wine contained a higher content of total anthocyanins, because of a significant content increase in the non-acetylated anthocyanins and the pyranoanthocyanins. This might be due to a concentration effect induced by the freeze concentration application to the grape must before fermentation. In addition, higher alcoholic strength in the treated wine might facilitate the extraction of anthocyanins during fermentation [52,53,54]. In our study, the pyranoanthocyanin content in the treated wine was about two times higher than that in the control. Pyranoanthocyanins have been reported to possess a more stable structure and have the capacity of enhancing the wine color by shifting wine color from red-purple to a more orange color [55]. Aging the wines for one-year significantly caused the content decrease of anthocyanins in these wines (Table 5), consistent with previous studies [42,56]. However, the treated wine still showed a higher content. Cold soak maceration application has also been reported to increase the content of anthocyanins in wine after fermentation. However, aging these wines for three months caused a dramatic decrease in the anthocyanin content, leading to a similar content as control [48]. Therefore, the freeze concentration treatment might be a better approach to stabilize anthocyanins in wine during aging. It was also noticed that aging resulted in a content percentage increase in the non-acetylated anthocyanins (from 65% to 74% approx.), but a decrease in the content percentage of the acetylated anthocyanins in the wines (from 31% to 25% approx.). Meanwhile, the percentage of the detected pyranoanthocyanins in the wine also decreased (from 4% to 2% approx.). These were not consistent with a previous report in which the pyranoanthocyanin content percentage increased after a 2-year aging period [42]. Further investigation should be carried out.

A significant difference in the color attributes was observed between the treated wine and the control after the fermentation process (Table 6). For example, the treated wine showed a lower value of L*, b*, and H, but a higher value of a* and C* compared with the control. This indicated that the freeze concentration treatment enhanced the red hue, and reduced the yellow hue of the wine. The enhancement of the red hue in the treated wine might be probably due to the higher amounts of anthocyanins [1,42], especially the non-acylated anthocyanins. The lightness was reduced after the freeze concentration treatment, whereas the chroma was improved. Aging the wines for one year significantly increased the value of b* and H (Table 6), indicating that these wines turned to be more yellowish in hue and their tone was much greater. The treated wine after one-year aging showed similar values of L*, a*, and C* to the control. However, its b* and H values were lower than the control, indicating that the control showed a more yellowish hue and greater tone than the treated wine. This indicated that the freeze concentration treatment had a much greater effect on the color enhancement of the fresh wine rather than the wine after the aging process.

### 2.4. Sensory Evaluation

**Figure 1:**
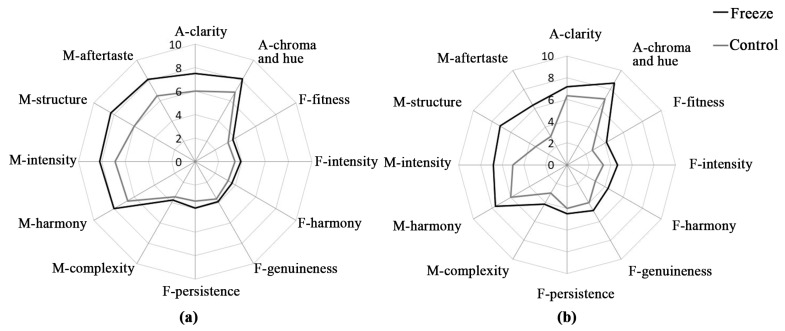
Organoleptic properties of freeze concentration treated wine and the control after (**a**) fermentation and (**b**) aging for one year. A, F, and M represent appearance, fragrance, and mouthfeel, respectively.

To further confirm the sensory feature differences that the freeze concentration process might bring to the wine, sensory evaluation of the two wines after the fermentation and the aging process, respectively, was carried out (Figure 1).

After fermentation, the treated wine showed a better sensory profile (Figure 1a). The obvious improvement was mainly highlighted in the appearance and mouthfeel, especially the structure and aftertaste properties. It has been confirmed that both the structure and the aftertaste of wine are mainly related to the composition and distribution of non-anthocyanin phenolic compounds [42,48]. In the present study, the freeze concentration treatment significantly changed the composition of flavan-3-ols and phenolic acids, which might result in such an alteration to the treated wine (Table 4). In addition, the improvement of the appearance in the treated wine could be mainly related to the higher level of anthocyanins, especially non-acylated anthocyanins (Table 4), since anthocyanins are the important contributor for wine color [57,58].

The fragrance also showed an improvement in the treated wine. As the results by GC-MS showed, the freeze concentration increased the contents of esters while it decreased the amount of volatile acids (*p* ≤ 0.05), which makes it a good way to improve wine aroma. It should be mentioned that although the treatment increased the contents of higher alcohols, it did not show a significant negative effect on wine aroma, which may be because the increased higher alcohols were still within the acceptable range as reported [23].

All things taken into consideration, the treated wine showed a better fragrance than the control according to the sensory panel (Figure 1a). Except for the composition of aromatic compounds, other factors could also play important roles in wine aroma [59]. For example, it has been reported that the synergistic effect from the low OAV value volatile compounds could improve the overall aroma of wine [34]. Similarly, the matrix effect of non-volatiles has been also reported to impact the wine aroma expression [7]. In the present study, glycerol had a higher content in the treated wine, which might enhance thje matrix effects. Differences in the sensory attributes of these wines after a one-year aging period were still observed (Figure 1b). For example, a significant improvement in the appearance of the treated wine still existed, which resulted mainly from the higher content of anthocyanins in the treated wine compared to the control. The scores on mouthfeel and fragrance properties decreased after the aging process, but fortunately the treated wine performed better than the control.

## 3. Materials and Methods

### 3.1. Vinification

Cabernet Sauvignon (*Vitis vinifera* L.) grape berries were harvested in September, 2014 in a commercial vineyard located at the Huojian Farm in Hami, Xinjiang, China (42°88′ N and 93°44′ E). The berries without physical injuries or infections were used for wine-making. After harvesting, the grape berries were crushed into must and 30 mg/L pectinase (EX-V, Lallemand Co., Toulous, France) and 40 mg/L SO_2_ were added

For the freeze concentration treated wine, 7000 L juice from the total 35,000 L was transferred into a 10,000 L cold stabilization tank. The juice was pumped into the scraper surface refrigerator (CR40000, VELO, Altivole, Italy). The freezing temperature was set at −10 °C. Then the frozen juice was pumped back to the cold stabilization tank, with circulation of this process for 24 h. Ice crystals were separated every 6 h. Approximately 3000 L concentrated juice was obtained. Concentrated juice was transferred to the primary tank and sufficient mixed with the remainder. Finally, a total of 31,000 L freeze concentrated must, with an increase of the pomace to juice ratio of approximately 12%, was used for further fermentation. Regarding the control, 35,000 L of the must without freezing was fermented directly.

The alcoholic fermentation was initiated after adding 150 mg/L 796 commercial *Saccharomyces cerevisiae* yeasts (Maurivin, Australia) in the tank and carried out at 26–28 °C for 7 days. After the alcoholic fermentation, the malolactic fermentation of the wine was immediately performed at 18 °C for 20 days. The fermentation was monitored by analyzing the L-malic acid consumption according to thin layer chromatography. After the malolactic fermentation was accomplished, the fresh wine was transferred into stainless steel tanks and aged for one year. The freeze concentration treated wine and the control were sampled after the malolactic fermentation and the aging process and they were analyzed and compared in terms of their aromatic and phenolic composition, color attributes, and sensory features, respectively. Each treatment was performed in duplicate.

### 3.2. Chemicals and Standards

Aromatic compound standards were purchased from Sigma-Aldrich (St. Louis, MO, USA). Phenolic compound standards were purchased from Shanghai Tauto Biotech Co. Ltd. (Shanghai, China). C_8_–C_21_ n-alkane series were purchased from Supelco (Bellefonte, PA, USA). Ethanol, methanol, formic acid, and acetonitrile were of HPLC grade and purchased from JandK Scientific Co. Ltd. (Beijing, China). Sodium hydroxide, sodium chloride, glucoside, and citric acid were obtained from Sinopharm Chemical Reagent Co. Ltd. (Beijing, China). Millipore water was purified from a Milli-Q system (Waters-Millipore Corporation, Bedford, MA, USA).

### 3.3. Standard Solution

Synthetic wine matrix was used to prepare standard solutions. The synthetic wine matrix, containing 13% ethanol (*v*/*v*), 2 g/L glucose, and 5 g/L citric acid, was adjusted pH to 3.8 using 5 M sodium hydroxide. A stock solution of volatile and phenolic compounds was prepared by dissolving them in ethanol and methanol, respectively, and then the working standard solutions were prepared by dissolving the stock solution using the synthetic wine matrix.

### 3.4. Volatile Compound Analyses

Volatile compound extraction and analysis followed a published method [15]. Briefly, 5 mL wine sample, 1 g sodium chloride, and 10 µL internal standard (IS, 4-methyl-2-pentanol, 1.0100 g/L in ethanol) were mixed into a 15 mL vial containing a magnetic stirrer. The vial was then sealed using a screw cap with a PTFE-silicon septum. Subsequently, the mixture in the vial was agitated on a magnetic plate (PC-400, Supelco, Bellefonte, PA, USA) at 40 °C for 30 min. Afterwards, a pre-conditioned DVB/CAR/PDMS solid phase microextraction (SPME) fiber (2 cm, 50/30 µm, Supelco, Munich, Germany) was inserted into the head space of the vial and the volatiles were absorbed for another 30 min under the same agitation condition. The desorption of the volatiles from the fiber was achieved by injecting the fiber into the GC injection port for 8 min at 250 °C. An Agilent 7890A gas chromatography (GC) coupled with an Agilent 5975C mass selective detector (MSD) was used for the analysis of the volatile compounds. An HP-INNOWAX capillary column (60 m × 0.25 mm i.d. × 0.25 μm film thickness, JandW Scientific, Folsom, CA, USA) was used for the separation of the volatiles. The carrier gas was helium and the flow rate was set at 1 mL/min. The oven temperature was programmed as follows: 50 °C kept for 1 min; 50–220 °C at a rate of 3 °C/min; 220 °C maintained for 5 min. The temperature of the detector and transfer line were set at 230 °C and 280 °C, respectively. The ion source was in electron impact (EI) mode at 70 eV. Full scan mode with the *m*/*z* range of 30 to 350 was used. Volatile compounds were identified and quantified on an off-line Agilent ChemStation Software (Agilent Technologies, Inc., Santa Clara, CA, USA). Identification was conducted by comparing their mass spectrum and retention indices (RIs) with the reference standards in the NIST11 library and further confirmed with their external standard. The volatile compounds without the external standards available were tentatively identified by comparing their RIs with the known pattern in the NIST11 database. For quantitation, the peak ratios of the target compound to the IS against the concentration were plotted to generate standard calibration curves. Compounds that had no standards were approximately quantified by the standards with the same functional group and/or similar amounts of carbon atoms [60].

### 3.5. Phenolic Compound Analyses

Non-anthocyanin phenolic compound analyses were performed on an Agilent 1260 Infinity Quaternary LC system coupled with an Agilent 6460 triple quadrupole mass spectrometer. The analysis conditions were set according to a previous published paper [61]. An Agilent Poroshell 120 EC-C18 column (3.0 × 50 mm, 2.7 µm) was used for the separation of the compounds. The wine sample was filtered through 0.22 µm membrane filters (Syringe Filter, PTFE, Supelco, Bellefonte, PA, USA), and 2 µL was then directly injected into the LC-MS system with a flow rate of 0.3 mL/min. The mobile phase was (A) water + 0.1% formic acid and (B) acetonitrile. The elution program was as follows: 0 to 1 min, 5% B; 1 to 3 min, 5% to 15% B; 3 to 15 min, 15% to 20% B; 15 to 18 min, 20% to 40% B; 18 to 20 min, 40% to 60% B; 20 to 20.1 min, 60% to 5% B; 20.1 to 30 min, 5% B. The column was maintained at 25 °C during the elution program. A total of 32 non-anthocyanin phenolic compound standards were collected based on the precursor to product ion transition using multiple reaction monitoring (MRM) mode under a negative electrospray ionization. These compounds in the wine sample were identified by comparing their mass spectrum and retention time with the external standard, and quantified using the external standard curve. Since the external standards, *cis*-piceid and *cis*-resveratrol, were not available, their content was quantified using their trans-isomer according to a published method [62].

The anthocyanin analyses were also carried out using the same apparatus with an Agilent Poroshell 120 EC-C18 column (3.0 × 100 mm, 2.7 µm) according to a previous method with minor modifications [62]. The wine sample was filtered through 0.22 µm membrane filters and 3 µL was injected. The flow rate was set at 0.3 mL/min and the column was maintained at 25 °C. The mobile phase was comprised of (A) 0.1% formic acid (*v*/*v*) in water and (B) 0.1% formic acid (*v*/*v*) in acetonitrile. The elution was programed as follows: 0 to 2 min, 8% B isocratic; 2 to 5 min, 8% to 15% B; 5 to 15 min, 15% to 25% B; 15 to 25 min, 25% to 70% B; 25 to 32 min, 70% to 8% B. A positive electrospray mode was used with the gas temperature of 330 °C, gas flow rate of 10 L/min, nebulizer pressure of 35 psi, and capillary voltage of 3500 V on the mass spectrometer. The mass spectrum analysis was conducted in multiple reaction monitoring (MRM) mode under a positive electrospray ionization. The quantitation of anthocyanins with the standard available was carried out using their external anthocyanin standards. The rest of the anthocyanins in the wine samples were quantified using the external standard that had the same anthocyanin aglycone.

### 3.6. Color Analysis

The color analysis (CIELa*b*) followed a published study [1]. The wine sample was transferred into a 2 mm path length glass cells and its absorbance was recorded at 420, 520, and 620 nm on a Shimadzu UV-2450 UV-Visible Spectrophotometer (Shimadzu Co. Ltd., Kyoto, Japan). The absorbance of the deionized water was used as the reference.

### 3.7. Sensory Evaluation

The sensory evaluation of the wine samples was performed based on a published method [9]. Briefly, a taste panel was selected among laboratory colleagues and winery technicians with more than 2 years of experience in wine sensory evaluation. The panel consisted of 15 sensory panelists (8 males and 7 females) with an age range from 25 to 45. All the panelists had acquired a training course on knowledge of wine sensory evaluation and were instructed on grade distribution and evaluating standards. In the evaluation form, the wine sensory attributes were graded using a 100 positive point method, including appearance (20 points in terms of clarity, chroma, and hue), fragrance (30 points regarding genuineness, intensity, fitness, harmony, and persistence), mouthfeel (40 points according to complexity, intensity, structure, harmony, and aftertaste), and overall impression (10 points). The evaluation was conducted in a standard sensory evaluation room. All samples were randomly marked with three-digit numbers and presented randomly. Between the samples, a 10 min break was given and the panelists were required to rinse their mouth with purified water.

### 3.8. Odor Activity Values

Odor activity values (OAV) refers to a ratio of the volatile concentration in wine and its odor threshold value and it is used to estimate the volatile contribution to wine aroma. A volatile compound with its OAV above 1 indicates its aroma contribution is significant. The OAV was calculated using the equation below.

OAV = Volatile Concentration/Odor Threshold

### 3.9. Statistical Analysis

Data were expressed as the mean ± standard deviation of duplicate tests. One-way analysis of variance with Duncan’s test was used to compare the differences between the means on the IBM S PSS 21.0 software package (SPSS Inc., Chicago, IL, USA). A difference of *p* ≤ 0.05 was considered as significant.

## 4. Conclusions

In conclusion, freeze concentration treatment elevated the level of alcohol and glycerol in wine, however, it did not change the other physicochemical properties of wine. This treatment led to higher amounts of esters as well as a lower volatile acids content, which could favor the fragrance quality of wine. Higher alcohols were increased by the treatment, but within an acceptable range, and they did not show a significant influence on the aroma character. Non-anthocyanin phenolic compositions showed differences between treated and control wines after fermentation. However, the differences disappeared after aging the wine for one year. A significant enhancement of the anthocyanin content was observed in the treated wine after fermentation and one-year aging. As a result, freeze concentration treatment significantly enhanced the wine color attributes, especially in the young wine. The treatment showed an improvement in appearance, fragrance, and mouthfeel, according to sensory evaluation. All results concluded that freeze concentration treatment of the must could be a good choice to improve the overall quality of wine.

## Figures and Tables

**Table 1:** Physicochemical properties of juices before and after freeze concentration treatment.

Physicochemical Index	Fresh	Concentrated	Mixed	Removed
Sugar (g/L)	228.23 ± 2.96 c	462.70 ± 9.21 a	255.71 ± 6.51 b	62.95 ± 5.43 d
Total acid (g/L)	5.44 ± 0.01 b	6.31 ± 0.22 a	5.44 ± 0.05 b	5.15 ± 0.17 b
Volatile acid (g/L)	0.08 ± 0.01 c	0.14 ± <0.01 a	0.10 ± <0.01 b	/
pH	3.78 ± 0.02 a b	3.74 ± 0.01 b	3.78 ± 0.03 a b	3.81 ± 0.01 a

Data are mean ± standard deviation of duplicate tests. Different letters in the same category indicate significant differences at *p* ≤ 0.05.

**Table 2:** Physicochemical properties of wines after fermentation and one-year aging.

Physicochemical Index	Fermentation		One-Year Aging	
	Control	Freeze Concentration	Control	Freeze Concentration
Alcohol (%, *v/v*)	13.32 ± 0.14 b	14.41 ± 0.18 a	13.12 ± 0.16 b	14.40 ± 0.16 a
Reducing sugar (g/L)	2.00 ± 0.14 a	1.45 ± 0.07 b	2.00 ± 0.14 a	2.35 ± 0.21 a
Total acid (g/L)	6.30 ± 0.28 a b	6.65 ± 0.35 a	6.00 ± 0.28 a b	5.85 ± 0.21 b
Volatile acid (g/L)	0.57 ± 0.02 b	0.55 ± 0.03 b	0.69 ± 0.03 a	0.71 ± 0.04 a
pH	3.82 ± 0.17 a	4.07 ± 0.18 a	3.97 ± 0.16 a	4.14 ± 0.16 a
Glycerol (g/L)	8.60 ± 0.28 b c	9.60 ± 0.28 a	8.00 ± 0.28 c	9.20 ± 0.28 a b

Data are mean ± standard deviation of duplicate tests. Different letters in the same category indicate significant differences at *p* ≤ 0.05.

**Table 3:** Content (μg/L), odor threshold (μg/L), and odor description of individual aromatic compounds in wine after fermentation and one-year aging.

Aromatic Compound	Threshold	Odor Description	Ref.	Fermentation		One-Year Aging	
				Control	Freeze Concentration	Control	Freeze Concentration
*Esters*							
**Ethyl acetate**	**12,000**	**pineapple, varnish, balsamic**	**[26]**	**47,948.68 ± 27.08 c**	**56,841.96 ± 1598.83 b**	**56,637.07 ± 2876.76 b**	**67,394.61 ± 797.76 a**
**Ethyl butanoate**	**20**	**strawberry, apple, banana**	**[27]**	**310.00 ± 24.08 a b**	**401.21 ± 59.90 a**	**315.73 ± 36.10 a b**	**214.16 ± 11.84 b**
**Isoamyl acetate**	**160**	**banana, fruity, sweet**	**[26]**	**1168.39 ± 32.31 b**	**1618.65 ± 58.36 a**	**770.28 ± 8.14 c**	**520.52 ± 14.98 d**
**Ethyl hexanoate**	**80**	**fruity, green apple, banana, brandy, winelike**	**[26]**	**383.38 ± 15.72 b**	**544.85 ± 26.57 a**	**398.17 ± 5.39 b**	**313.98 ± 14.31 c**
Hexyl acetate	1500	fruity, pear	[28]	4.74 ± 0.35 b	6.71 ± 0.25 a	2.92 ± 0.28 c	1.76 ± 0.21 d
Ethyl heptanoate *	300	winelike, brandy, fruity	[29]	0.64 ± 0.06 b	0.92 ± 0.12 a	0.60 ± 0.08 b	1.11 ± 0.02 a
Ethyl lactate	14,000	fruity, buttery	[15,30]	2136.21 ± 175.76 b	2520.77 ± 22.81 a	2034.38 ± 1.17 b	2374.45 ± 184.53 a b
Isobutyl hexanoate *				4.17 ± 0.31 b	5.77 ± 0.14 a	3.27 ± 0.06 c	3.16 ± 0.27 c
Methyl octanoate	200	intense citrus	[31]	2.81 ± 0.13 a	2.5 ± 0.19 a	2.50 ± 0.14 a	1.61 ± 0.06 b
**Ethyl octanoate**	**580**	**sweet, floral, fruity, banana, pear, brandy**	**[26]**	**616.91 ± 27.32 a**	**696.89 ± 49.33 a**	**607.38 ± 40.03 a**	**438.71 ± 7.53 b**
Isoamyl hexanoate	1000	Pineapple, cheese	[28]	5.25 ± 0.21 a b	5.52 ± 0.11 a	5.19 ± 0.08 a b	5.03 ± 0.01 b
Propyl octanoate *				2.78 ± 0.19 a	2.72 ± 0.21 a	3.49 ± 0.55 a	1.44 ± 0.10 b
Ethyl nonanoate	1300	Rose, fruity	[28]	2.25 ± 0.12 a	1.76 ± 0.04 a	2.65 ± 0.97 a	2.54 ± <0.01 a
Ethyl 2-hydroxy-4-methyl pentanate *				12.59 ± 0.17 c	10.31 ± 0.40 c	42.14 ± 1.51 b	50.19 ± 1.00 a
Isobutyl octanoate *	800		[28]	5.25 ± 0.52 a b	5.92 ± 0.33 a	4.81 ± 0.40 b	3.08 ± 0.21 c
**Isoamyl lactate**	**200**	**buttery, nut**	**[30]**	**119.12 ± 12.93 d**	**157.74 ± 4.28 c**	**320.45 ± 7.38 b**	**372.32 ± 7.52 a**
Methyl decanoate *	1200		[28]	3.03 ± 0.28 a b	2.83 ± 0.18 a b	3.51 ± 0.45 a	2.32 ± 0.08 b
**Ethyl decanoate**	**200**	**grape, fruit**	**[27]**	**284.87 ± 10.63 b**	**335.91 ± 5.92 a**	**298.78 ± 31.9 a b**	**235.01 ± 2.79 c**
Isoamyl octanoate	1000	Sweet, light fruity, cheese, cream	[28]	37.14 ± 0.83 b	43.37 ± 1.41 a	33.83 ± 2.73 b	24.83 ± 1.16 c
Ethyl benzoate	575		[25]	0.82 ± 0.01 b	0.35 ± 0.04 c	1.29 ± 0.07 a	0.75 ± 0.01 b
Diethyl succinate	6000	overripe melon, lavender, vinous	[26]	161.95 ± 20.7 c	275.01 ± 36.86 c	843.63 ± 54.47 b	1185.03 ± 158.12 a
Ethyl 9-decenoate	100	green, fruity, fatty	[32]	6.19 ± 0.78 a b	28.78 ± 16.12 a	4.56 ± 0.19 b	7.54 ± 1.20 a b
Propyl decanoate *				0.45 ± 0.03 b	0.62 ± 0.10 a	nd	nd
Ethyl undecanoate *				0.62 ± 0.06 a	0.71 ± 0.06 a	0.49 ± 0.29 a	0.30 ± <0.01 a
Methyl salicylate	40		[33]	3.90 ± 0.15 c	2.12 ± 0.01 c	24.46 ± 1.61 a	12.52 ± 0.68 b
Ethyl phenylacetate		rose, floral	[20]	1.24 ± 0.06 c	1.11 ± <0.01 c	1.81 ± 0.06 b	3.53 ± 0.07 a
Ethyl salicylate				11.64 ± <0.01 b	11.32 ± 0.02 c	11.86 ± 0.04 a	11.81 ± 0.04 a
Phenethyl acetate	1800	fruity	[26]	33.82 ± 0.55 a	33.61 ± 2.40 a	24.50 ± 0.35 c	28.06 ± 0.23 b
Ethyl dodecanoate	500	fruity, floral	[29]	58.37 ± 4.40 a b	68.87 ± 4.29 a	47.41 ± 7.66 b c	34.97 ± 2.25 c
Isopentyl decanoate *				33.42 ± 5.48 b	50.44 ± 0.10 a	30.2 ± 6.91 b	23.56 ± 3.73 b
Ethyl dihydrocinnamate *	**1.6**	**sweet, pleasant**	**[34]**	**3.17 ± 0.08 a**	**nd**	**3.33 ± 0.22 a**	**2.36 ± 0.05 b**
Ethyl myristate *	500	mild waxy, soapy	[29]	2.22 ± 0.39 a	1.09 ± 0.11 b	1.71 ± 0.42 a b	1.35 ± 0.13 b
Ethyl palmitate *	1000	mild waxy	[29]	1.65 ± 0.35 a	1.45 ± 0.13 a	1.32 ± 0.41 a	1.08 ± 0.01 a
*Total* (mg/L)				*53.37 ± 0.06 c*	*63.68 ± 1.75 b*	*62.48 ± 3.07 b*	*73.27 ± 1.17 a*
*Alcohols*							
1-Propanol	306,000	alcohol, ripe fruit	[26]	53,963.28 ± 818.91 b	63,554.43 ± 2075.83 a	55,066.05 ± 3306.10 b	54,425.82 ± 856.53 b
Isobutanol	75,000	alcohol, nail polish	[26]	46,796.40 ± 388.87 b	50,229.69 ± 1074.77 a b	46,078.47 ± 2717.74 b	51,738.37 ± 1485.45 a
1-Butanol	150,000	medicinal, phenolic	[26]	1564.43 ± 32.26 b	1511.50 ± 39.64 b	1731.64 ± 62.71 a	1740.55 ± 8.08 a
**Isopentanol**	**60,000**	**nail polish, alcohol**	**[26]**	**240,695.90 ± 197.47 b**	**274,678.10 ± 9597.97 a**	**242,018.97 ± 7302.91 b**	**291,089.97 ± 8184.10 a**
1-Pentanol	64,000	almond, balsam	[4]	106.36 ± 10.08 a	109.19 ± 5.01 a	102.24 ± 6.20 a	109.62 ± 5.33 a
4-Methyl-1-pentanol	50,000	almond, toasty	[4]	63.73 ± 3.10 b	71.55 ± 1.17 a	61.89 ± 0.80 b	75.73 ± 1.75 a
3-Methyl-1-pentanol	50,000	vinous, herbaceous, cacao	[4]	157.40 ± 0.62 a b	165.77 ± 1.48 a	151.50 ± 0.28 b	156.37 ± 6.72 a b
**1-Hexanol**	**1100**	**herbaceous**	**[26]**	**2554.32 ± 32.42 c**	**3221.72 ± 100.59 a**	**2774.28 ± 48.47 b**	**3048.03 ± 52.86 a**
(*E*)-3-Hexen-1-ol	400	green, floral	[31]	73.70 ± 2.73 b c	92.23 ± 3.61 a	80.61 ± 3.08 c	85.94 ± 0.79 a b
(*Z*)-3-Hexen-1-ol	400	green	[31]	40.89 ± 3.57 b	39.80 ± 3.85 b	71.16 ± 2.25 a	67.32 ± 0.52 a
3-Octanol *				0.96 ± 0.04 a b	0.84 ± <0.01 b	1.02 ± 0.06 a	0.99 ± 0.07 a
(*E*)-2-Hexen-1-ol	400	green grass, herb	[31]	nd	nd	52.09 ± 1.31 a	50.46 ± 0.76 a
1-Octen-3-ol	20	mushroom	[15]	4.72 ± 0.14 b	3.50 ± 0.14 c	7.69 ± 0.60 a	7.85 ± 0.35 a
2-Ethyl-1-hexanol	8000	mushroom, sweet fruity	[31]	0.47 ± 0.16 c	1.01 ± 0.04 b c	1.14 ± 0.33 b	6.38 ± 0.17 a
3-Ethyl-4-methyl pentanol *				94.31 ± 1.03 c	82.69 ± 2.16 c	144.34 ± 6.14 b	284.78 ± 11.70 a
2-Nonanol	58	coconut	[31]	1.16 ± 0.04 b	0.95 ± 0.04 c	1.75 ± 0.06 a	1.74 ± 0.08 a
1-Octanol	800	jasmine, lemon	[26]	21.24 ± 0.23 b	24.14 ± 0.37 a	25.06 ± 1.07 a	23.59 ± 0.09 a
1-Nonanol *	600		[30]	45.30 ± 0.65 c	47.00 ± 1.50 b c	50.72 ± 2.20 b	68.54 ± 1.09 a
(*Z*)-6-Nonen-1-ol				3.39 ± 0.01 a	2.57 ± 0.30 b	3.58 ± 0.27 a	3.75 ± 0.34 a
Methionol	1500	cooked potato, garlic	[26]	1366.57 ± 12.59 a b	918.91 ± 10.76 c	1277.70 ± 105.32 b	1503.03 ± 21.60 a
1-Decanol	500	floral, fruity, waxy, fatty	[29]	4.45 ± 0.04 a b	3.54 ± 0.18 c	4.87 ± 0.40 a	3.94 ± 0.02 b c
Benzyl alcohol	900,000	roasted, toasted	[26]	664.65 ± 7.16 b	625.89 ± 26.81 b	2227.26 ± 232.54 a	2635.85 ± 194.02 a
**2-Phenylethanol**	**14,000**	**rose, honey**	**[15]**	**39,049.57 ± 149.55 a**	**34,466.48 ± 625.96 c**	**39,525.08 ± 428.78 a**	**36,755.50 ± 1107.36 b**
1-Dodecanol	1000	unpleasant odor, fruity	[32]	28.82 ± 0.71 a b	17.93 ± 1.21 c	35.00 ± 5.52 a	24.77 ± 0.27 b c
*Total* (mg/L)				*387.30 ± 8.66 b*	*429.87 ± 9.39 a*	*391.49 ± 7.59 b*	*443.91 ± 10.20 a*
*Acids*							
Acetic acid	200,000	sour, pungent, vinegar	[27]	835.24 ± 59.97 b	586.37 ± 40.95 c	1053.23 ± 140.12 a	1101.65 ± 126.00 a
Propanoic acid	8100	pungent, rancid, soy	[15]	1288.69 ± 10.23 a	467.10 ± 60.68 b	1233.23 ± 167.68 a	166.30 ± 0.82 c
Isobutyric acid	2300	pungent, rancid, soy	[15]	629.42 ± 43.37 a	682.41 ± 10.66 a	647.43 ± 4.71 a	626.32 ± 38.08 a
Isovaleric acid	3000	rancid	[15]	648.75 ± 73.56 c	807.17 ± 3.61 b	540.34 ± 47.71 c	1049.51 ± 18.05 a
**Hexanoic acid**	**420**	**rancid, cheese**	**[27]**	**1758.46 ± 59.97 a b**	**1658.15 ± 49.67 b**	**2053.87 ± 143.66 a**	**1302.42 ± 160.19 c**
**Octanoic acid**	**500**	**rancid, cheese, fatty**	**[27]**	**1118.91 ± 12.80 b**	**799.53 ± 9.29 c**	**1556.45 ± 112.66 a**	**789.14 ± 36.13 c**
Decanoic acid	1000	rancid, fatty	[27]	576.75 ± 56.04 b	471.12 ± 58.04 c	850.88 ± 38.43 a	472.15 ± 4.68 c
*Total* (mg/L)				*6.86 ± 0.16 b*	*5.47 ± 0.21 c*	*7.94 ± 0.58 a*	*5.51 ± 0.37 c*
*Terpenes*							
β-Myrcene	15	fruity, grape, wine-like	[33,35]	0.87 ± 0.01 a	0.83 ± <0.01 a	0.86 ± 0.07 a	0.84 ± 0.03 a
Linalool	15	citrus, floral, sweet, grape-like	[26]	1.16 ± 0.03 c	1.27 ± 0.03 c	1.98 ± 0.12 b	2.56 ± 0.06 a
α-Terpineol	250	lilac, floral, sweet	[15]	0.71 ± 0.03 b	1.26 ± 0.39 a b	1.11 ± 0.07 a b	1.41 ± 0.02 a
β-Citronellol	100	rose	[26]	6.47 ± 0.08 a b	6.59 ± 0.12 a	6.17 ± 0.12 b	5.54 ± 0.16 c
**Geraniol**	**30**	**roses, geranium**	**[4]**	**124.61 ± 10.75 a b**	**147.46 ± 6.45 a**	**107.33 ± 14.81 b c**	**82.84 ± 7.74 c**
**Nerol**	**15**	**orange flowers, rose**	**[27]**	**36.89 ± 1.10 a**	**38.28 ± 2.95 a**	**35.75 ± 0.01 a**	**37.21 ± 0.35 a**
*Total* (µg/L)				*170.70 ± 11.69 a b*	*195.86 ± 8.86 a*	*153.18 ± 15.07 b c*	*130.39 ± 7.15 c*
*C13-norisoprenoids*							
**β-Damascenone**	**0.14**	**floral, stewed apple**	**[15]**	**0.75 ± 0.04 a**	**0.77 ± 0.02 a**	**0.64 ± 0.13 a b**	**0.49 ± 0.05 b**
Geranylacetone	60	flora	[31]	0.71 ± 0.05 b	1.08 ± 0.08 a	0.77 ± 0.11 b	0.66 ± 0.01 b
*Total*				*1.46 ± 0.01 b*	*1.85 ± 0.10 a*	*1.40 ± 0.24 b*	*1.16 ± 0.04 b*
*Aldehydes*							
Nonanal	1	herbaceous	[36]	0.29 ± 0.13 a	0.48 ± 0.04 a	0.45 ± 0.57 a	0.90 ± 0.09 a
Furfural	14,100	toasty	[31]	tr	tr	20.66 ± 1.17 b	137.98 ± 1.85 a
Decanal	1000	grassy, orange skin	[31]	1.91 ± 0.02 b	1.87 ± 0.04 b	2.13 ± 0.29 b	3.74 ± 0.06 a
Benzealdehyde	2000	toasty, almond	[31]	19.84 ± 0.25 c	17.85 ± 0.04 d	20.58 ± 0.09 b	21.24 ± 0.31 a
Dodecanal				3.51 ± 0.01 a	tr	3.52 ± 0.06 a	tr
*Total* (µg/L)				*25.54 ± 0.42 c*	*20.2 ± 0.05 c*	*47.32 ± 0.34 b*	*163.85 ± 2.32 a*
*Volatile phenols*							
Phenol	25,000	phenolic	[37]	39.66 ± 0.97 b	19.83 ± 1.52 d	51.37 ± 3.42 a	27.59 ± 0.37 c
*Others*							
Styrene *				7.33 ± 0.26 b	4.66 ± 0.18 c	6.88 ± 0.21 c	4.97 ± 0.06 a
Acetoin	150,000	buttery, fatty	[26]	5845.39 ± 95.08 b	798.1 ± 4.56 c	1338.62 ± 413.99 c	11,827.10 ± 956.3 a
Naphthalene *				1.10 ± 0.03 b	1.34 ± 0.01 a	1.15 ± 0.01 b	1.33 ± 0.04 a

Data are mean ± standard deviation of duplicate tests. Compounds were quantified using their corresponding standard; Compounds labeled with * were quantified using the compounds listed in brackets: Ethyl heptanoate and Ethyl 2-hydroxy-4-methyl pentanate (Ethyl hexanoate), Ethyl undecanoate and Ethyl dihydrocinnamate and Ethyl myristate and Isopentyl decanoate (Ethyl dodecanoate), Ethyl palmitate (Ethyl dodecanoate), Isobutyl hexanoate and Isobutyl octanoate (Ethyl octanoate), Propyl octanoate and Propyl decanoate (Ethyl nonanoate), Methyl decanoate (Ethyl decanoate), 1-Nonanol and 3-Octanol and 3-Ethyl-4-methyl pentanol (1-Octanol), β-Damascenone (β-Lonone), Styrene (Phenol) and Naphthalene (Phenol). Compounds with OAV above 1.0 are highlighted in bold font. Different letters in the same category indicate significant differences at *p ≤* 0.05. “tr” and “nd” represent “trace amount” and “not detected”, respectively. The odor thresholds and odor descriptions are obtained from the references listed in the ‘Ref.’ Column.

**Table 4:** Content (mg/L) of individual non-anthocyanins in wine after fermentation and one-year aging.

Non-Anthocyanin	Fermentation		One-Year Aging	
	Control	Freeze Concentration	Control	Freeze Concentration
*Flavan-3-ols*				
Catechin	46.91 ± 5.39 b	64.70 ± 6.28 a	40.44 ± 4.16 b	38.44 ± 2.90 b
Epicatechin	12.79 ± 1.02 b	18.21 ± 3.09 a	12.86 ± 1.10 b	12.89 ± 1.56 b
Procyanidin B2	16.27 ± 0.22 a	15.10 ± 0.58 a	14.62 ± 2.20 a	17.66 ± 1.93 a
Procyanidin B1	13.41 ± 1.05 a	9.43 ± 0.97 b	14.22 ± 1.69 a	14.00 ± 1.47 a
(−)-Gallocatechin	2.53 ± 0.11 a	2.67 ± 0.33 a	2.20 ± 0.24 a	2.19 ± 0.26 a
(−)-Epigallocatechin	2.36 ± 0.44 a	1.74 ± 0.34 a	2.05 ± 0.34 a	1.59 ± 0.16 a
Epicatechin gallate	0.24 ± 0.01 c	1.28 ± 0.22 a	0.60 ± 0.10 b	0.22 ± 0.01 c
*Total*	*94.51 ± 8.02 b*	*113.13 ± 9.56 a*	*87.00 ± 9.83 b*	*87.00 ± 8.26 b*
*Flavonols*				
Quercetin-3-*O*-glucuronide	13.13 ± 1.5 a	11.06 ± 1.42 a	9.97 ± 1.25 a	10.71 ± 1.13 a
Quercetin-3-*O*-glucoside	5.26 ± 0.30 a	5.94 ± 0.41 a	2.67 ± 0.58 b	2.69 ± 0.30 b
Quercetin	7.03 ± 0.79 b	2.97 ± 0.19 c	9.54 ± 0.71 a	10.04 ± 1.23 a
Myricetin	2.99 ± 0.43 b	2.32 ± 0.17 b	4.42 ± 0.45 a	5.55 ± 0.60 a
Isorhamnetin-3-*O*-glucoside	1.55 ± 0.13 a b	1.71 ± 0.2 a	1.24 ± 0.28 a b	1.04 ± 0.16 b
Quercetin-3-*O*-galactoside	1.76 ± 0.17 a	1.68 ± 0.22 a	1.19 ± 0.09 b	0.90 ± 0.13 b
Quercetin-3-*O*-rutinoside	nd	1.37 ± 0.23 a	0.12 ± 0.01 b	0.09 ± 0.01 b
Kaempferol-3-*O*-glucoside	0.64 ± 0.09 b	1.16 ± 0.09 a	0.20 ± 0.01 c	0.19 ± 0.01 c
Quercetin-3-*O*-rhamnetin	nd	0.32 ± 0.04 a	0.31 ± 0.04 a	0.36 ± 0.05 a
Taxifolin	0.49 ± 0.06 a	0.31 ± 0.05 b	0.37 ± 0.04 a b	0.45 ± 0.03 a
Isorhamnetin	0.21 ± 0.03 c	0.07 ± 0.01 d	0.34 ± 0.02 b	0.42 ± 0.02 a
Kaempferol	tr	tr	0.19 ± 0.02 b	0.38 ± 0.06 a
*Total*	*33.05 ± 3.00 a*	*28.93 ± 2.47 a*	*30.55 ± 3.20 a*	*32.83 ± 3.69 a*
*Phenolic acids*				
Gallic acid	16.57 ± 0.95 a	13.98 ± 1.96 b	4.91 ± 0.47 c	4.63 ± 0.50 c
Syringic acid	2.12 ± 0.21 b	2.75 ± 0.31 a	1.62 ± 0.32 b	2.15 ± 0.04 b
Gentisic acid	0.96 ± 0.08 b	1.29 ± 0.05 a	0.09 ± 0.02 c	0.13 ± 0.01 c
Caffeic acid	3.22 ± 0.20 c	0.96 ± 0.09 d	8.90 ± 0.15 a	6.77 ± 0.35 b
Vanillic acid	0.48 ± 0.05 a b	0.53 ± 0.06 a	0.30 ± 0.05 c	0.36 ± 0.06 b c
Ferulic acid	0.17 ± 0.01 c	0.18 ± 0.02 c	0.76 ± 0.06 b	0.98 ± 0.04 a
*p*-Hydroxybenzoic acid	0.03 ± <0.01 c	0.05 ± <0.01 b	0.05 ± <0.01 b	0.09 ± 0.01 a
*p*-Coumaric acid	0.25 ± 0.02 b	0.03 ± <0.01 c	1.41 ± 0.21 a	1.62 ± 0.17 a
*Total*	*23.79 ± 1.51 a*	*19.76 ± 1.76 b*	*18.03 ± 0.96 b*	*16.72 ± 1.05 b*
*Stilbenes*				
*cis*-Piceid	1.62 ± 0.26 a	1.38 ± 0.09 a	0.76 ± 0.16 b	0.95 ± 0.06 b
*trans*-Piceid	1.08 ± 0.03 a	0.93 ± 0.15 a	0.16 ± 0.01 c	0.49 ± 0.06 b
*cis*-Resveratrol	1.77 ± 0.32 a	1.83 ± 0.13 a	1.39 ± 0.09 b	0.57 ± 0.03 c
*trans*-Resveratrol	0.95 ± 0.14 a b	1.14 ± 0.06 a	0.71 ± 0.13 b	0.42 ± 0.04 c
Picentannol	0.34 ± 0.03 a	0.17 ± 0.02 b	0.10 ± 0.01 c	0.14 ± 0.02 b c
*Total*	*5.76 ± 0.79 a*	*5.46 ± 0.07 a*	*3.12 ± 0.38 b*	*2.57 ± 0.06 b*

Data are mean ± standard deviation of duplicate tests. Different letters in the same row indicate significant differences at *p* ≤ 0.05. tr and nd represent trace amount and not detected, respectively.

**Table 5:** Content (mg/L) of individual anthocyanins in wine after fermentation and one-year aging.

Anthocyanin	Quant **	Fermentation		One-Year Aging	
		Control	Freeze Concentration	Control	Freeze Concentration
*Non-acylated anthocyanins*					
Delphinidin-3,5-*O*-diglucoside	I	1.15 ± 0.09 c	1.78 ± 0.11 a	1.18 ± 0.13 c	1.43 ± 0.11 b
Peonidin-3,5-*O*-diglucoside	II	0.09 ± 0.01 b	0.15 ± 0.01 a	0.09 ± 0.01 b	0.10 ± 0.01 b
Delphinidin-3-*O*-glucoside	III	15.50 ± 1.20 b c	27.34 ± 1.58 a	10.52 ± 0.82 d	12.81 ± 1.18 cd
Malvinidin-3,5-*O*-diglucoside *	IV	32.44 ± 2.16 a	tr	tr	18.78 ± 3.70 a
Cyanidin-3-*O*-glucoside	V	2.29 ± 0.17 b	3.24 ± 0.27 a	1.42 ± 0.16 c	2.39 ± 0.21 b
Petunidin-3-*O*-glucoside	IV	40.27 ± 4.16 b	48.07 ± 2.58 a	19.01 ± 1.35 c	22.40 ± 1.50 c
Peonidin-3-*O*-glucoside	II	9.06 ± 0.86 a	8.36 ± 0.50 a b	5.05 ± 0.32 c	7.57 ± 0.48 b
Malvinidin-3-*O*-glucoside	IV	95.00 ± 1.72 a	93.78 ± 4.84 a	62.02 ± 2.99 c	74.32 ± 4.37 b
*Total*		*163.40 ± 4.77 b*	*182.73 ± 6.17 a*	*99.29 ± 3.07 d*	*121.04 ± 3.90 c*
*Acylated anthocyanins*					
Delphinidin-3-*O*-(6-*O*-acetyl)-glucoside	III	6.45 ± 2.96 b	10.21 ± 0.64 a	4.41 ± 0.35 b	4.92 ± 0.30 b
Cyanidin-3-*O*-(6-*O*-acetyl)-glucoside	V	3.45 ± 0.29 a	3.39 ± 0.30 a	1.33 ± 0.10 b	1.62 ± 0.17 b
Petunidin-3-*O*-(6-*O*-acetyl)-glucoside	IV	16.07 ± 1.29 a	16.25 ± 1.67 a	6.60 ± 0.43 b	7.93 ± 0.45 b
Peonidin-3-*O*-(6-*O*-acetyl)-glucoside	II	7.16 ± 0.67 a	5.80 ± 0.34 b	2.32 ± 0.26 c	3.02 ± 0.29 c
Malvinidin-3-*O*-(6-*O*-acetyl)-glucoside	IV	40.87 ± 3.01 a	43.51 ± 2.63 a	16.82 ± 1.08 b	21.11 ± 1.37 b
*cis*-Delphinidin-3-*O*-(6-*O*-coumaryl)-glucoside	III	1.13 ± 0.08 a	0.67 ± 0.05 b	0.21 ± 0.01 c	0.21 ± 0.02 c
*trans*-Delphinidin-3-*O*-(6-*O*-coumaryl)-glucoside *	III	78.40 ± 8.18 a	36.16 ± 1.96 b	12.63 ± 0.77 c	16.32 ± 1.42 c
*cis*-Petunidin-3-*O*-(6-*O*-coumaryl)-glucoside *	IV	71.12 ± 8.36 a	82.83 ± 9.20 a	16.11 ± 2.10 b	12.49 ± 2.86 b
*trans*-Petunidin-3-*O*-(6-*O*-coumaryl)-glucoside	IV	1.51 ± 0.09 a	0.93 ± 0.07 b	0.25 ± <0.01 a	0.27 ± 0.02 b
Peonidin-3-*O*-(6-*O*-caffeoyl)-glucoside *	II	38.93 ± 2.98 a	20.79 ± 1.18 b	5.78 ± 0.53 c	10.13 ± 0.67 c
Malvidin-3-*O*-(6-*O*-caffeoyl)-glucoside *	IV	107.63 ± 12.23 a	90.46 ± 5.50 a	21.42 ± 4.13 b	31.28 ± 1.90 b
*cis*-Peonidin-3-*O*-(6-*O*-coumaryl)-glucoside *	II	85.06 ± 7.23 a	46.15 ± 3.96 b	19.38 ± 1.20 c	19.97 ± 1.56 c
*trans*-Peonidin-3-*O*-(6-*O*-coumaryl)-glucoside	II	1.61 ± 0.19 a	0.92 ± 0.07 b	0.31 ± 0.02 c	0.35 ± 0.02 c
Malvinidin-3-*O*-(6-*O*-coumaryl)-glucoside	IV	5.91 ± 0.32 a	5.02 ± 0.45 b	1.49 ± 0.12 c	1.59 ± 0.15 c
*Total*		*84.54 ± 2.38 a*	*86.97 ± 4.17 a*	*33.82 ± 0.65 c*	*41.10 ± 1.29 b*
*Pyranoanthocyanins*					
Petunidin-3-*O*-glucoside-pyruvate	IV	0.17 ± 0.02 a	0.15 ± 0.02 a	0.14 ± 0.01 a	0.17 ± 0.01 a
Malvinidin-3-*O*-glucoside-pyruvic acid	IV	0.95 ± 0.07 a	0.84 ± 0.05 a b	0.69 ± 0.05 b c	0.86 ± 0.06 a
Malvinidin-3-*O*-glucoside-acetaldehyde	IV	1.07 ± 0.11 b	6.78 ± 0.39 a	0.80 ± 0.07 b c	0.30 ± 0.02 c
Peonidin-3-*O*-(6-*O*-acetyl)-glucoside-4-vinylphenol *	II	61.39 ± 6.9 a	47.36 ± 3.17 b	31.28 ± 4.40 c	41.12 ± 2.28 b c
Malvinidin-3-*O*-(6-*O*-acetyl)-glucoside-pyruvic acid	IV	0.42 ± 0.03 a	0.34 ± 0.02 b	0.23 ± 0.02 c	0.31 ± 0.03 b
Malvinidin-3-*O*-(6-*O*-acetyl)-glucoside-acetaldehyde	IV	0.26 ± 0.01 b	2.37 ± 0.31 a	0.21 ± 0.02 b	0.07 ± <0.01 b
Malvinidin-3-*O*-(6-*O*-coumaryl)-glucoside-acetaldehyde *	VI	50.07 ± 3.68 b	309.55 ± 20.28 a	30.80 ± 1.58 b c	12.17 ± 1.09 c
Petunidin-3-*O*-glucoside-4-vinylphenol *	IV	93.07 ± 7.62 a	16.50 ± 1.60 b	45.31 ± 3.10 a	28.18 ± 1.80 a b
Malvinidin-3-*O*-glucoside-vinylguaiacol *	IV	45.77 ± 5.04 a	7.15 ± 0.37 c	20.22 ± 2.17 b	17.71 ± 0.97 b
Malvinidin-3-*O*-glucoside-4-vinylphenol	IV	0.78 ± 0.09 a	0.16 ± 0.01 c	0.45 ± 0.02 b	0.29 ± 0.02 c
Peonidin-3-*O*-(6-*O*-acetyl)-glucoside-4-viniylphenol *	II	36.28 ± 3.49 a	5.80 ± 1.08 b	8.83 ± 1.05 b	6.50 ± 0.59 b
Malvinidin-3-*O*-(6-*O*-acetyl)-glucoside-4-vinylphenol *	IV	157.31 ± 16.91 a	36.10 ± 4.01 b	56.89 ± 4.67 b	39.14 ± 4.24 b
Malvinidin-3-*O*-(6-*O*-coumaryl)-glucoside-vinylphenol *	IV	26.36 ± 4.64 a	3.55 ± 0.32 b	8.55 ± 0.93 b	5.22 ± 0.41 b
*Total*		*4.11 ± 0.25 b*	*11.05 ± 0.58 a*	*2.71 ± 0.05 c*	*2.15 ± 0.07 c*
*Polymeric anthocyanin*					
(epi)catechin-ethyl-Malvidin-3-*O*-glucoside isomer 1 *	IV	4.94 ± 0.35 b	8.71 ± 1.12 a	1.18 ± 0.03 c	0.84 ± 0.10 c
(epi)catechin-ethyl-Malvidin-3-*O*-glucoside isomer 2 *	IV	12.18 ± 1.56 a	12.70 ± 1.13 a	2.17 ± 0.13 b	1.52 ± 0.14 b
(epi)catechin-ethyl-Malvidin-3-*O*-glucoside isomer 3 *	IV	12.42 ± 0.12 a	9.79 ± 0.54 b	3.61 ± 0.14 c	3.09 ± 0.17 c
(epi)catechin-ethyl-Malvidin-3-*O*-glucoside isomer 4 *	IV	14.40 ± 0.67 b	20.32 ± 0.84 a	1.98 ± 0.13 c	1.31 ± 0.13 c
*Total*		*0.04 ± <0.01 a*	*0.05 ± <0.01 b*	*0.01 ± <0.01 c*	*0.01 ± <0.01 c*

Data are mean ± standard deviation of duplicate tests. Different letters in the same row indicate significant differences at *p* ≤ 0.05. Compounds labelled with * are quantified in µg/L, whereas the rest of the compounds are quantified in mg/L. ** represents the quantification category of the compounds: I, delphinidin-3,5-*O*-diglucoside; II, peonidin-3-*O-*glucoside; III, delphinidin-3-*O-*glucoside; IV, malvidin-3-*O-*glucoside; and V, cyanidin-3-*O-*glucoside.

**Table 6:** Color attributes of wine after fermentation and one-year aging.

Color Attribute	Fermentation		One-Year Aging	
	Control	Freeze Concentration	Control	Freeze Concentration
L*	65.52 ± 3.70 a	52.37 ± 3.05 b	61.61 ± 3.93 a	62.64 ± 3.83 a
a*	37.3 ± 2.09 b c	49.37 ± 3.10 a	32.69 ± 1.72 c	36.98 ± 2.19 b c
b*	5.97 ± 0.47 c	3.61 ± 0.20 d	19.32 ± 1.44 a	13.40 ± 0.97 b
C*	37.78 ± 0.22 b	49.51 ± 0.11 a	37.97 ± 0.04 b	39.34 ± 0.19 b
H	9.10 ± 0.67 c	4.18 ± 0.16 d	30.59 ± 0.88 a	19.91 ± 0.15 b

Data are mean ± standard deviation of duplicate tests. Different letters in the same row indicated significant differences at *p* ≤ 0.05.
